# Endophytic Fungi from Frankincense Tree Improves Host Growth and Produces Extracellular Enzymes and Indole Acetic Acid

**DOI:** 10.1371/journal.pone.0158207

**Published:** 2016-06-30

**Authors:** Abdul Latif Khan, Ahmed Al-Harrasi, Ahmed Al-Rawahi, Zainab Al-Farsi, Aza Al-Mamari, Muhammad Waqas, Sajjad Asaf, Ali Elyassi, Fazal Mabood, Jae-Ho Shin, In-Jung Lee

**Affiliations:** 1 UoN Chair of Oman’s Medicinal Plants & Marine Natural Products, University of Nizwa, Nizwa, Oman; 2 School of Applied Biosciences, Kyungpook National University, Daegu, Republic of Korea; 3 Department of Agriculture, Abdul Wali Khan University Mardan, Mardan, Pakistan; Estación Experimental del Zaidín (CSIC), SPAIN

## Abstract

*Boswellia sacra*, an economically important frankincense-producing tree found in the desert woodlands of Oman, is least known for its endophytic fungal diversity and the potential of these fungi to produce extracellular enzymes and auxins. We isolated various fungal endophytes belonging to Eurotiales (11.8%), Chaetomiaceae (17.6%), Incertae sadis (29.5%), Aureobasidiaceae (17.6%), Nectriaceae (5.9%) and Sporomiaceae (17.6%) from the phylloplane (leaf) and caulosphere (stem) of the tree. Endophytes were identified using genomic DNA extraction, PCR amplification and sequencing the internal transcribed spacer regions, whereas a detailed phylogenetic analysis of the same gene fragment was made with homologous sequences. The endophytic colonization rate was significantly higher in the leaf (5.33%) than the stem (0.262%). The Shannon-Weiner diversity index was *H*′ 0.8729, while Simpson index was higher in the leaf (0.583) than in the stem (0.416). Regarding the endophytic fungi’s potential for extracellular enzyme production, fluorogenic 4-methylumbelliferone standards and substrates were used to determine the presence of cellulases, phosphatases and glucosidases in the pure culture. Among fungal strains, *Penicillum citrinum* BSL17 showed significantly higher amounts of glucosidases (62.15±1.8 μM^-1^min^-1^mL) and cellulases (62.11±1.6 μM^-1^min^-1^mL), whereas *Preussia* sp. BSL10 showed significantly higher secretion of glucosidases (69.4±0.79 μM^-1^min^-1^mL) and phosphatases (3.46±0.31μM^-1^min^-1^mL) compared to other strains. *Aureobasidium* sp. BSS6 and *Preussia* sp. BSL10 showed significantly higher potential for indole acetic acid production (tryptophan-dependent and independent pathways). *Preussia* sp. BSL10 was applied to the host *B*. *sacra* tree saplings, which exhibited significant improvements in plant growth parameters and accumulation of photosynthetic pigments. The current study concluded that endophytic microbial resources producing extracellular enzymes and auxin could establish a unique niche for ecological adaptation during symbiosis with the host Frankincense tree.

## Introduction

**Fig 1:**
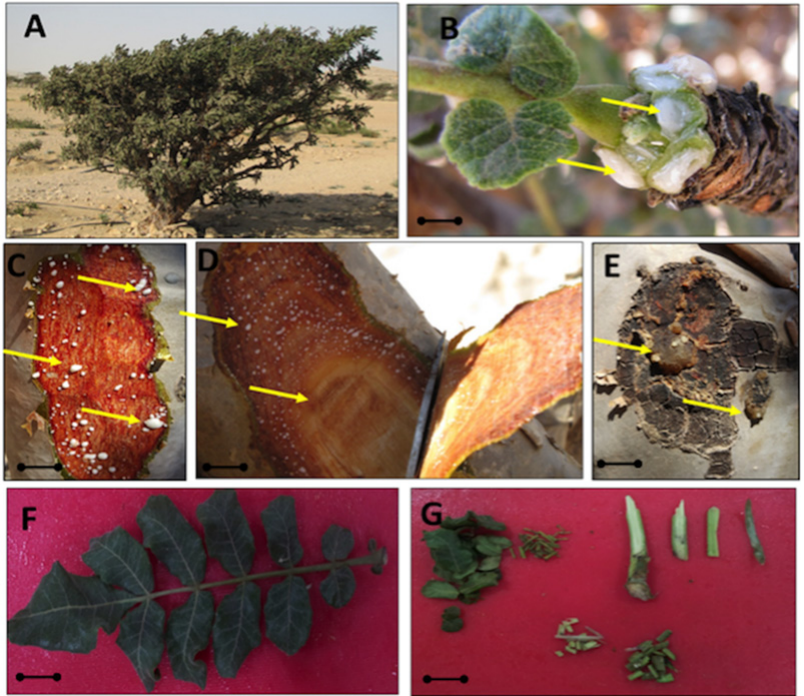
Sample collection and processing for the isolation of fungal endophytes from the frankincense (*Boswellia sacra*) tree. (**A**) Shows the hot and dry habitat of the frankincense tree; (**B**) resin emerging from the mature leaf of tree branches; (**C**) the milk-like resin oozing out from the lower epidermal layer of the tree; (**D**) the inner bark or the cortex region of the tree did not contain resinous ducts and was also evaluated for the presence of endophytes; (**E**) dried wounded outer bark in which the white milk was converted into crystalline gum (**F**); leaf pattern of the frankincense tree; (**G**) smaller parts of the tree (leaf, stem and bark parts) were used for isolation. A total of 208 10-mm tissue samples from the leaf and stem (bark) were used to isolate endophytic fungi. The black line is equivalent to 1 inch.

*Boswellia sacra* is one of the economically important *frankincense-* or *olibanum-*producing trees of the Sultanate of Oman. Resin from *Boswellia* has been traded as incense from the southern coast of Arabia to the Mediterranean region for more than a millennium [1]. There are about twenty species of *Boswellia*, and *Boswellia sacra* is an endemic specie that grows only in the Dhofar region of Oman. It is a keystone specie that is known to provide an important oleoresin gum. The resin has strong cultural and medicinal value. The essential oil and boswellic acid or its derivatives from the resin have been known to possess potent anticancer activities [2]. The local population obtains solid and semi-solid resin (commonly known as *Luban*) by making a series of wounds/incisions in the bark of the tree (Fig 1). The annual production of Omani frankincense ranges between 80 to 100 tons from nearly 500,000 trees [3]. In some areas, the collection of resin is an economically favorable use of land than crop production and accounts for the majority of a rural household’s income [4].

The tree activates its physiological defense mechanism by producing volatile and non-volatile chemical messengers to counteract the negative impacts of resin production that occurs when wounded. A series of anthropogenically-induced wound/incisions can activate both primary and secondary defense responses in trees [5–6]. In addition to the plant’s own natural defense mechanisms, various types of symbiotic microorganisms also play a crucial role in plant life. Among microorganisms, endophytes have been regarded as essential, playing a crucial role in plant defense responses [7].

Endophytes are ubiquitous microorganisms (fungi or bacteria) that live inside plant tissues without causing any symptoms of disease. Endophytic microorganisms have been known to exist in symbiosis with hosts throughout their life cycle, starting from seed germination until fruit development [8]. The failure to exploit endophytic fungi depends on our limited understanding of the evolutionary significance of these organisms and their dynamic interaction with their respective hosts. During the long period of co-evolution, a friendly relationship has been formed between each endophyte and its host plant [9]. The plant provides a protective sanctuary and access to nutrients, while in return, the endophyte produces bioactive secondary metabolites and enzymes [10–12]. These secondary metabolites can range from various types of phenolics to hormone-like compounds. In the last decade, reports have shown that the various classes of endophytic fungi produce auxin (indole acetic acid) and gibberellins (GA_3_, GA_4_ and GA_7_) [13–16].

Besides production of plant growth-promoting hormones, endophytes have also been found to produce various types of extracellular enzymes such as phosphatase, zylanase, cellulases etc [17–20]. Secretion of such enzymes along with phytohormone-like compounds, can extend greater benefits to the host plant during normal and stressful environmental conditions [9, 21]. Assessing and identifying symbiotic microbial diversity of economically important and ecologically unique endemic tree species helps in the understanding of the growth and development of the host plants. The Frankincense tree (*Boswellia sacra*) can grow in extremely arid environmental conditions, where water and nutrient availability are at their lowest levels. In such circumstances, elucidating the endophytic fungal diversity of the tree can help in understanding the tree’s life and co-evolution with symbionts, and assessing the bioactive potentials of those endophytes can pave the way for their eco-friendly application as inoculants. Since *B*. *sacra* is very difficult to propagate due to its harsh nature, utilizing endemic microflora as plant growth promoter can help in increasing the declining population of the tree [22]. Hibbett et al. [23] suggested that this level of diversity could include more than 8.7 million species in both rhizosphere and phyllosphere. However, the global diversity of these endophytic fungi is limitless, and a large number of species and ecosystems need to be explored.

In this regards, much attention has been given to the exploring the microorganisms in the rhizospheric region of the tree compared to the phyllospheric or aerial part of the tree. Aerial plant (phylloplane (leaf) and caulosphere (stem) is a primary key habitat for many microorganisms and plays an important role with governing processes happening at the interface between atmosphere, plants and microorganisms [16]. Similarly, a significant amount of work has also been carried out on the endophytes derived from the forest ecosystem [24]; however, the arid land ecosystem is frequently overlooked. Although the resin-based chemical constituents of *Boswellia sacra* and their role in human health have been extensively studied [25], almost nothing is known about the microbial diversity and extracellular enzyme production of this tree. Additionally, the effects of isolated endophytic fungi on the host tree are relatively unknown. Therefore, the present study aimed to explore endophytic fungal diversity, the potential of these fungi to produce extracellular enzymes and indole acetic acid. The potentially bioactive endophyte was applied to assess its role in improvement of host plant growth as well.

## Materials and Methods

### Ethics Statement

The study sites were managed by local tree owners in Wadi Dawkah. However, we collected samples from the trees that grew wildly in the area, where no specific permit was required for taking samples. Our study did not use any endangered or protected species. The trees used for sampling were treated ethically, and our study did not harm the local environment.

### Site description and plant growth conditions

Wadi Dawkah, Dhofar-Oman (17°25ʹ21ʹʹN; 54°00ʹ32ʹʹE) belongs to a completely arid desert region with small sandstone hills. The annual mean temperature of the sampling area is ~35°C, and the annual rainfall is approximately 60 mm. The temperature in summer also reaches ~47°C. Parts of the of *Boswellia sacra* plant, such as the leaf and stem, were collected (Fig 1) from 10 different trees. The *B*. *sacra* tree grows in the desert woodlands (Fig 1A) with meager amounts of water and low nutrient availability [3, 26]. Most wild or partially domesticated trees are supplied with water [27]. The whitish milk resin oozes out from inner epidermal regions, while the axillary buds and leaf emerging axes also produce resin milk (Fig 1B and 1C). However, in response to a deep incision (10–15 mm) in the inner cortex (parenchyma and sclerenchyma) regions, we observed fewer or no milk production sites. This suggests a lack of resinous ducts at such depths (Fig 1D). After wounding, the milky resin takes 3 to 5 days to solidify (Fig 1E).

### Plant sample collection and endophyte isolation

To isolate endophytes, 30 healthy trees (1.4 m) were selected for sampling. These trees were growing wildly in Wadi Dawkah without traces of recent tapping for resin extraction. Bark and leaf part of the trees were collected and brought to the laboratory in sterilized (autoclaved at 121°C for 15 min) zipper bags in an ice box (4°C). The samples were cut into 0.5 cm pieces to differentiate the outer and inner bark (Fig 1G). The samples were surface sterilized with sodium hypochlorite (2.5%; 30 min in a shaking incubator at 120 rpm) and repeatedly washed with autoclaved distilled water (DDW) to remove any epiphytic microbes and ecto-mycorrhizae [13,28]. Isolation of fungi from the bark/leaves were carried out on Hagem minimal medium, containing 0.5% glucose, 0.05% KH_2_PO_4_, 0.05% MgSO_4_.7H_2_O, 0.05% NH_4_Cl, 0.1% FeCl_3_, 100 ppm streptomycin and 1.5% agar (pH 5.8 ±0.2). The newly emerged fungal spots were separated and further grown and stored on potato dextrose agar (PDA, streptomycin 50 ppm). The efficiency of sterilization was monitored by imprinting the tissues on Hagem and PDA plates. Upon contaminant growth, the tree samples were sterilized again. The morphologically different [24] endophytic fungal strains were grown in Czapek broth medium (1% Glucose, 1% Peptone, 0.05% KCl, 0.05% MgSO_4_.7H_2_O, and 0.001% FeSO_4_.7H_2_O; pH 7.3±0.2) and incubated on shaking incubator (28°C with 150 rpm for 8 days).

### Molecular microbial identification

The endophytic microbes were grouped into different morpho-types based on colony shape, thickness, color of aerial hyphae, colony reverse color, growth rate and pattern, margin characteristics, surface texture, and growth depth into medium [24]. The endophytes were identified by genomic DNA (gDNA) extraction, PCR techniques, nucleotide sequencing, and phylogenetic analysis as described by Khan et al. [14]. gDNA was isolated according to manufacturing instructions from fresh mycelial mates with a genomic DNA preparation kit (Solgent, Korea). The isolated fungi were identified by sequencing the nuclear ribosomal internal transcribed region (ITS) using the following universal primers: ITS-1, 5′-TCCGTA GGT GAA CCT GCG G-3′ and ITS-4, 5′-TCC TCC GCT TAT TGA TAT GC-3′ [28–29]. The online BLASTn search pipeline was used to compare the nucleotide sequence similarity of the isolated strains with homologues ITS regions of related fungi. Additional sequences were downloaded from nucleotide repository of GenBank in MEGA version 6.0 [30]. All sequences were aligned using pair-wise CLUSTALW alignment [30]. The missing or ambiguous sequences were either coded as N or deleted [29]. The phylogenetic tree of all the aligned sequences were made using neighbor-joining, maximum parsimony and maximum likely-hood trees in MEGA 6.0 software. Two thousand bootstrap replications were used as a statistical support for the nodes in the phylogenetic tree. Descriptive tree statistics, tree length, consistency index, rescaled consistency index, retention index, and homoplasy index were calculated for Most Parsimonious Tree (MPT) generated [30–31]. The aligned sequence data was submitted to GenBank for accession number.

### Quantification of extracellular enzymes

To quantify extracellular enzymes, the method of Marx et al. [32] was adopted with some modifications. Briefly, all the substrates (S1 Table and S1 Fig) were obtained from Sigma-Aldrich Co. Ltd in crystalline form. Ten milliliters of a 10 mM stock solution of each 4-methylumbelliferone (MUB) substrate was prepared, while the assay procedures were the same for each substrate. Depending on the substrate, a 7-MUB standard was used. A 10 mM stock solution of pure MUB was prepared in methanol (0.1762 g of 4-methylumbelliferone in 100 mL). This stock solution was diluted in MES buffer to 1 μM and stored at 4°C (S1 Table).

The endophytic fungi grown in potato dextrose broth were harvested using centrifugation (4°C, 12,000 rpm for 10 min). The pure and fresh culture filtrates (CF) were syringe filtered (0.22 μm) to remove traces of turbidity. For each type of enzyme analysis, a minimum of three replicates for each substrate (CF + buffer + substrate), a quenched standard (sample + buffer + 4-MUB), and a substrate control (media + buffer + substrate) were maintained. The total volume of liquid in the cuvette was 2 mL CF or buffer or media and 100 μL substrate or 4-MUB with different types of CF obtained from endophytic fungi. The pre-optimized fluorescence spectrophotometer (Shimadzo, Tokyo, Japan) was used to read the absorbance with 360 nm excitation and 460 nm emission at time zero and 30-minute intervals for 2 hours. The readings were calculated according to this formula:

Activity (μmol h^-1^ L^-1^) = slope of concentration versus time in hours

$$\text{Enzymes concentrations}=\frac{\mathrm{Substrate}-\mathrm{substrate control}}{\frac{\mathrm{Standard}-\mathrm{sample blank}}{\mathrm{concentration standard}\times \mathrm{volume standard}}\times \mathrm{volume culture filtrate}}$$

### Indole Acetic Acid quantification by UPLC-MS/MS

The estimation of the level of indole-3-acetic acid (IAA) in the culture broth was performed using colorimetric assay as shown by Hoffman et al. [33]. All the isolated endophytes were cultured in 20 mL Czapek broth with and without L-tryptophan for seven days (incubated at 30 ± 2°C; 200 rpm). The cell-free cultures obtained after centrifugation (10,000 × *g* for 10 min at 4°C) were filtered through a 0.45-μm cellulose acetate filter (DISMIC^®^, Denmark). 1 N HCl was used to acidify the cell-free cultures (pH 2.8) and subsequently extracted 3 times with 20 mL ethyl acetate. The extracted fractions were combined prior to evaporate under a vacuum at 45°C in a rotary evaporator. The resultant residue was re-suspended in 3 mL 50% methanol: water and one mL of it was mixed with 2 mL Salkowski reagent (12 g FeCl_3_/L of 7.9 M H_2_SO_4_) for one hour in dark condition. Readings for change in color was noted at 535 nm in ELISA Spectrophotometer (xMark BioRad, USA). The IAA in culture broth was quantified against separately prepared standard IAA (Sigma-Aldrich, Korea). A total of five replications (100 ml in Erlenmeyer flask) were used to make sure the validation of IAA results.

After finding positive results for endophytes, we selected one of the bioactive strains that showed maximum IAA production in the colorimetric assay for further quantification with UPLC as described in the method of Khan et al. [34] (UPLC-ESI-MS/MS conditions mentioned in S2 Table). Two external standard preparations (IAA dissolved in 100% water) and their average response factor was used for the quantification of IAA. Therefore the area under the peak for each Multiple Reaction Monitoring (MRM) trace was integrated and obtained by the transition of the precursor ion (175.65) to the product ion (129.8) for both the standards and the samples. The purity of standard (98% w/w) was also considered to calculate response factor.

### Endophytic fungus application to the host tree and growth analysis

The tree saplings of four-week-old *Boswellia sacra*, which originated from seeds, were donated by the Museum of Frankincense Land, Ministry of Heritage & Culture, Salalah, Sultanate of Oman. They were grown in a mix of peat moss, clay and sand (1:1:8) to enable sufficient aeration. The bioactive endophyte (BSL10) was selected on the basis of its potential to produce essential enzymes and IAA for plant-microbe interaction experiment [35]. The endophyte was grown in Czapek Broth (autoclaved 500 mL media supplemented with 100 ppm streptomycin) as mentioned in the endophyte isolation section for 14 days in incubator shaker (150 rpm, 30 ±2°C). The endophytic mycelial cells along with pure culture were applied three times to the four-week-old saplings (7, 14 and 21 days after treatment—DAT). The first readings (0 DAT) sought to understand the dynamics of growth promotion by BSL10 compared to control. The control plants only received fungus-free media. After three weeks of endophytic inoculation (21 DAT), plant growth promoting parameters (numbers of leaves, internodes and shoot lengths) were recorded. The chlorophyll content (*a*, *b* and total carotenoids) was determined using the method of Khan and Lee [13]. Each treatment included nine replications.

### Statistical Analysis

The colonization density, colonization rates and isolation rates of fungal diversity were calculated as the percentage of segments infected by one or more isolates from the total number of segments of each plated tissue sample using the method of Kumar and Hyde [35]. Shannon-Wiener and Simpson diversity indices were calculated for isolated endophytic fungi from *Boswellia sacra* through the online system (http://www.alyoung.com/labs/biodiversity_calculator.html). Species richness was also calculated using the same webpage, and a rarefaction curve was formed. All samples were analyzed in triplicate. The data are presented as the mean ± standard error of the mean (SEM). Differences were evaluated using one-way analysis of variance (ANOVA). Differences were considered significant at *P* < 0.05 and were calculated by GraphPad Prism Version 6.01 for Windows (GraphPad Software, San Diego, CA, USA). The mean values were compared using Duncan’s multiple range tests at *P* < 0.05 (SAS 9.1, Cary, NC, USA). Unscrambler version 9.0 by Camo was used for principal component analysis (PCA) to determine the correlation of enzyme production abilities by the endophytic fungi. The PCA model was built with three different extracellular enzymes—glucosidases, phosphatases and cellulases—and a full cross-validation was used to validate the enzyme production ability among different species.

## Results

### Endophyte diversity with the *Boswellia sacra* tree

Endophytic fungi were isolated from different organs (leaf and stem) of *Boswellia sacra*. A total of 30 trees were selected for sample collection and approximately 120 stem and 185 leaf samples were sterilized for endophytic fungal isolation. These tissue segments resulted in the isolation of 77 isolates. Seventeen fungal endophytes were selected (Table 1 and S2 Fig), which were grouped based on morphological trait analysis as suggested by Arnold et al. [24]. However, the overall analysis suggested that 17 endophytic fungi possessed different morphological characteristics, while some were found to be telemorphs of each other. The colonization rate was also significantly higher in the leaves of *B*. *sacra* compared to the stems. The colonization rate for the leaf was 4.08%, while that of the stem was 2.03%. Similarly, the isolation rate was significantly higher in leaves (5.33%) compared to stems (0.262%). The results showed that more than 50% of the fungi were from leaves, 38% were from stems. Rarefaction indices were employed to compare the richness of endophytic fungi in *B*. *sacra*, and the level of the Shannon-Weiner diversity index was high (*H*′ = 0.8729). The Simpson index (1/*l*) was the highest for leaves (0.583), while it showed a very low level of species richness in stems (0.416) through the dominance index. These endophytic fungi were identified by molecular methods.

**Table 1:** Endophytic fungal strains isolated from the stem and leaf parts of the Frankincense tree.

Strain ID	Name	Isolated from	BLASTn Homology (%)	Accession number (GenBank)
BSL11	*Chaetomium* sp.	Leaf	99	KX233834
BSL10	*Preussia* sp.	Leaf	99	KR231682
BSL21	*Penicillium citrinum*	Leaf	99	KX233837
BSS7	*Thielavia arenaria*	Leaf	98	KR231678
BSS14	*Phoma medicaginis*	Stem	98	KR231679
BSS6	*Aureobasidium *sp.	Stem	98	KX233836
BSL12	*Preussia* sp.* *	Leaf	99	KR231680
BSS11	*Dothideomycetes* sp.* *	Leaf	99	KR231681
BSS1-2b	*Aureobasidium pullulans*	Stem	98	KX233835
BSS1	*Phoma* sp.	Leaf	93	KR231683
BSL17	*Penicillium citrinum*	Leaf	98	KR231677
BSL13	*Aureobasidium pullulans*	Stem	98	KX233838
BSS15	*Aureobasidium pullulans*	Stem	98	KX233839
BSPDB	*Thielavia arenaria*	Leaf	99	KX233840
L2	*Sordariomycetes* sp.	Leaf	98	KX233841
B3	*Fusarium proliferatum*	Stem	97	KX233842
BSL2-1	*Preussia* sp.	Leaf	95	KX233846

### Identification of endophytic fungi and phylogenetic analysis

**Fig 2:**
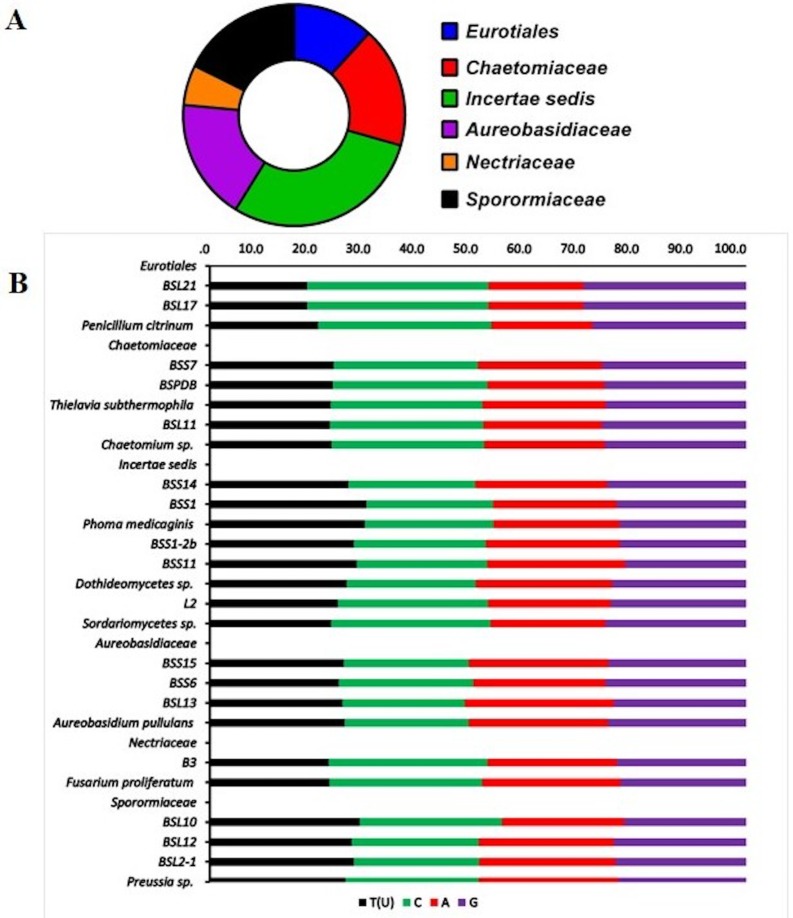
Endophytic distribution and nucleotide homology. Endophyte isolated from *B*. *sacra*, family-wise distribution (A) and comparison of nucleotide composition in the sequence with those from related strains in GenBank (B). A comparative assessment was made with the help of MEGA 6.0 [30].

**Fig 3:**
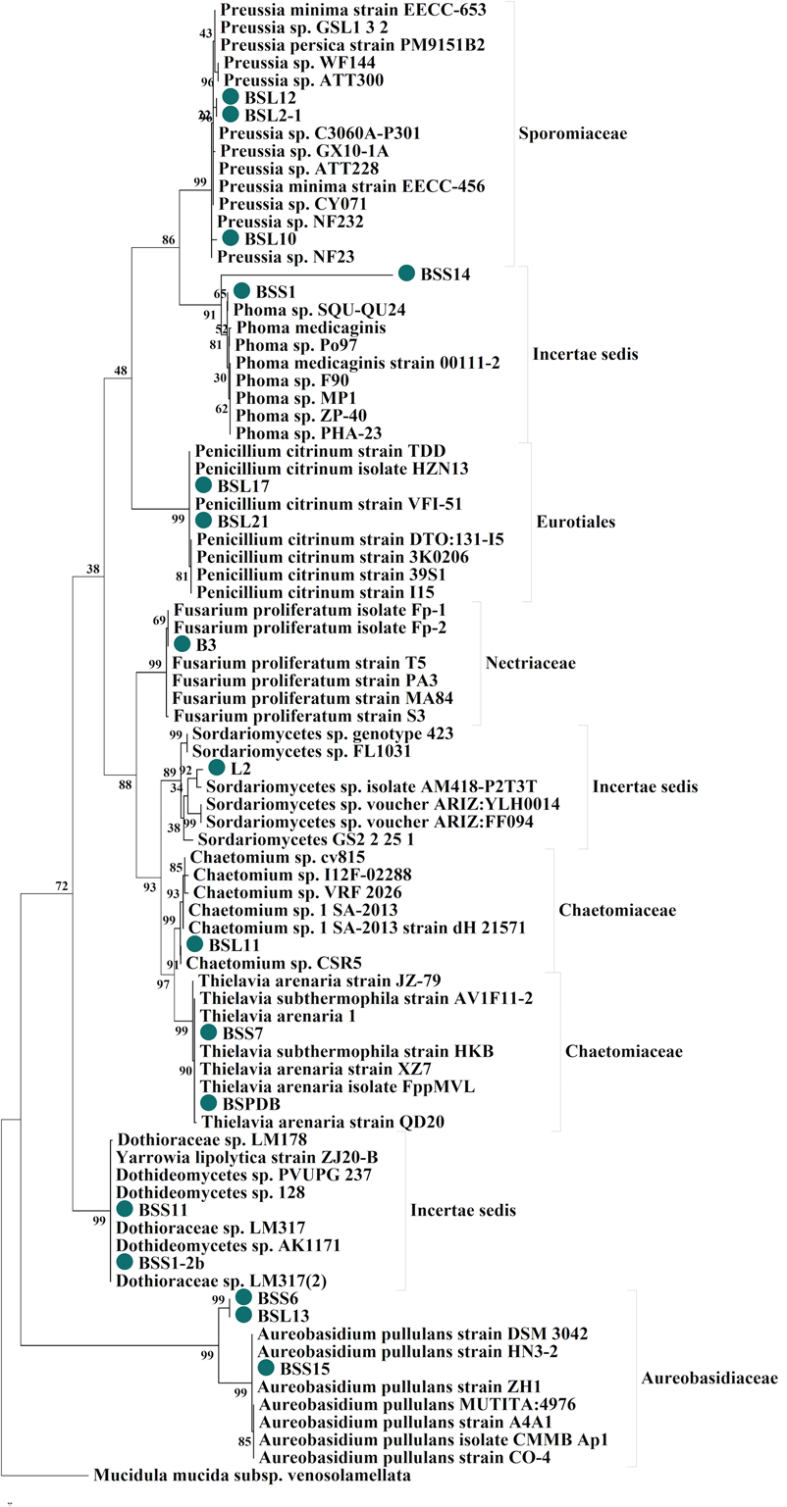
Phylogenetic analysis of endophytes. Maximum Parsimony analysis of isolated endophytes based on the sequences obtained from the sequencing process of the internal transcribed spacer (ITS) region. The phylogeny was constructed using the homologous fungal sequences deposited in GenBank. The percentage of replicate trees in which the associated species clustered together in the bootstrap test (2000 replicates) are shown next to the branches. The analysis involved 84 nucleotide sequences. All positions containing gaps and missing data were eliminated. There were a total of 296 positions in the final dataset. Evolutionary analyses were conducted in MEGA 6.0 [30].

The ITS sequences of the rRNA gene region from the 17 fungi revealed that the fragment lengths ranged from 518–630 bp. The average T(U), C, A, and G ratio was 24.5, 26.5, 23.6 and 25.3 respectively (Fig 2). We isolated various fungal endophytes belonging to Eurotiales (11.8%), Chaetomiaceae (17.7%), Incertae sadis (29.4%), Aureobasidiaceae (17.7%), Nectriaceae (5.9%) and Sporomiaceae (17.7%) from the bark and leaf parts of the tree (Fig 2). The sequences were aligned in MEGA 6.0 and BLASTn searched to correlate them with the highly homologous fungal strains. Most of the fungal sequences showed 95–100% homology with related fungi (Table 1). Based on this 95–100% sequence similarity, we identified the fungal strains as species of *Chaetomium* sp., *Preussia* sp. (3 strains), *Penicillium citrinum* (2 strains), *Thielavia arenaria* (2 strains), *Phoma* sp. (2), *Aureobasidium* sp. (3 strains), and *Dothideomycetes* sp. (2 strains), *Sordariomycetes* sp. and *Fusarium proliferatum* (Table 1 and Fig 3). The phylogenetic analysis of these strains showed 95–99% homology with ITS sequences of rRNA genes of related species (Fig 3). The evolutionary history was inferred using the Maximum Parsimony method. For all sites of the parsimony-informative indexes such as consistency index (CI = 0.66), retention index (RI = 0.95), and composite index (CI = 0.65) was also noted (Fig 3). The overall branch length in the phylogenetic tree was 3.9 and 4.06 in neighbor joining and UPGMA methods with 2K bootstrap replications [30]. In case of maximum likelihood analysis, estimated Transition/Transversion bias (R) was 0.96 and a log value of -3016.078 was noted for the 84 sequences with 296 positions in final dataset. The average evolutionary divergence of overall sequence pairs was 0.69±0.05. The sequences were deposited in the NCBI GenBank to obtain accession numbers as shown in Table 1.

### Extracellular enzyme quantification from endophytes

To detect whether the isolated endophytic fungi had the ability to produce extracellular enzymes during axenic conditions, all of the strains were grown in broth medium for 7 days and centrifuged to obtain pure fungal-free culture. Fluorescence-based MUB standards were used to analyze the presence of three enzymes (β-1,4-glucosidase, 1,4-β-cellobiosidase, and phosphatase; S1 Table). Standard curve readings were taken for the excitation and emission of the fluorogenic substrate in the presence of buffer and in combination with MUB standard (S1 Fig). Curve fit values for cellulases (R^2^ = 0.98), phosphatases (R^2^ = 0.96) and glucosidases (R^2^ = 0.98) were recorded using a fluorescence spectrophotometer.

The different fungal strains showed varying concentrations of cellulases, phosphatases and glucosidases in the pure culture filtrates (Table 2). Different isolates of similar strains also showed significant variations in the concentrations of these enzymes. *Preussia* sp., *Phoma medicaginis* and *Penicillium citrinum* had significantly higher concentrations of glucosidases. Phosphatase production was significantly higher in *Preussia* sp., and the other strain of *Preussia* sp. also significantly exhibited similarly high enzyme production. *Thielavia microspore* and *Penicillium citrinum* produced significantly more cellulase enzymes compared to other strains and species of fungi (Table 2).

**Table 2:** Extracellular enzymes production ability of endophytes isolated from the different part of Frankincense tree.

Strain ID	Name	Glucosidase (μM^-1^min^-1^mL)	Phosphatases (μM^-1^min^-1^mL)	Cellulases (μM^-1^min^-1^mL)
BSL11	*Chaetomium* sp.	0.37±0.00h	0.21±0.001e	0.55±0.15g
BSL10	*Preussia* sp.	69.4±0.79a***	3.46±0.31a**	0.66±0.004g
BSL21	*Penicillium citrinum*	45.33±0.05d	2.29±0.00b	0.41±0.001g
BSS7	*Thielavia arenaria*	55.14±1.75c	0.40±0.01e	55.14±1.75b*
BSS14	*Phoma medicaginis*	62.57±2.45b***	0.32±0.007e	9.20±0.01e
BSS6	*Aureobasidium *sp.	35.49±1.59e	0.27±0.008	35.49±1.59c
BSL12	*Preussia* sp.* *	2.21±1.10g	2.91±0.16b	2.21±1.107f
BSS11	*Dothideomycetes* sp.* *	45.96±2.6d	0.68±0.02d	45.96±2.6b
BSS1-2b	*Aureobasidium pullulans*	43.62±1.2d	0.68±0.02d	45.96±2.61b
BSS1	*Phoma* sp.	56.07±0.94c	0.31±0.03e	9.14±0.07e
BSL17	*Penicillium citrinum*	62.15±1.8b***	0.59±0.05d	62.15±1.86a***
BSL13	*Aureobasidium pullulans*	9.71±0.23f	1.7±0.06c	50.2±0.70b
BSS15	*Aureobasidium pullulans*	6.13±0.81fg	0.60±0.03d	33.1±0.86c
BSPDB	*Thielavia arenaria*	6.83±0.21fg	0.70±0.03d	32.7±1.06c
L2	*Sordariomycetes* sp.	9.14±0.27f	0.31±0.17d	56.07±0.39b
B3	*Fusarium proliferatum*	3.95±0.94g	0.71±0.31d	29.17±0.75d
BSL2-1	*Preussia* sp.	3.80±0.42g	2.88±0.21b	27.23±0.92d

The values with * (*P*<0.05)

** (*P*<0.01) and

*** (*P*<0.001) shows a significant difference in the overall capacity to produce extracellular enzymes among endophytic fungi.

The different letters in each column shows that different fungal samples are producing significantly different concentrations of extra-cellular enzymes in the culture. The different letter shows a variation in mean values among different groups, which was calculated by the DMRT analysis using SAS software.

**Fig 4:**
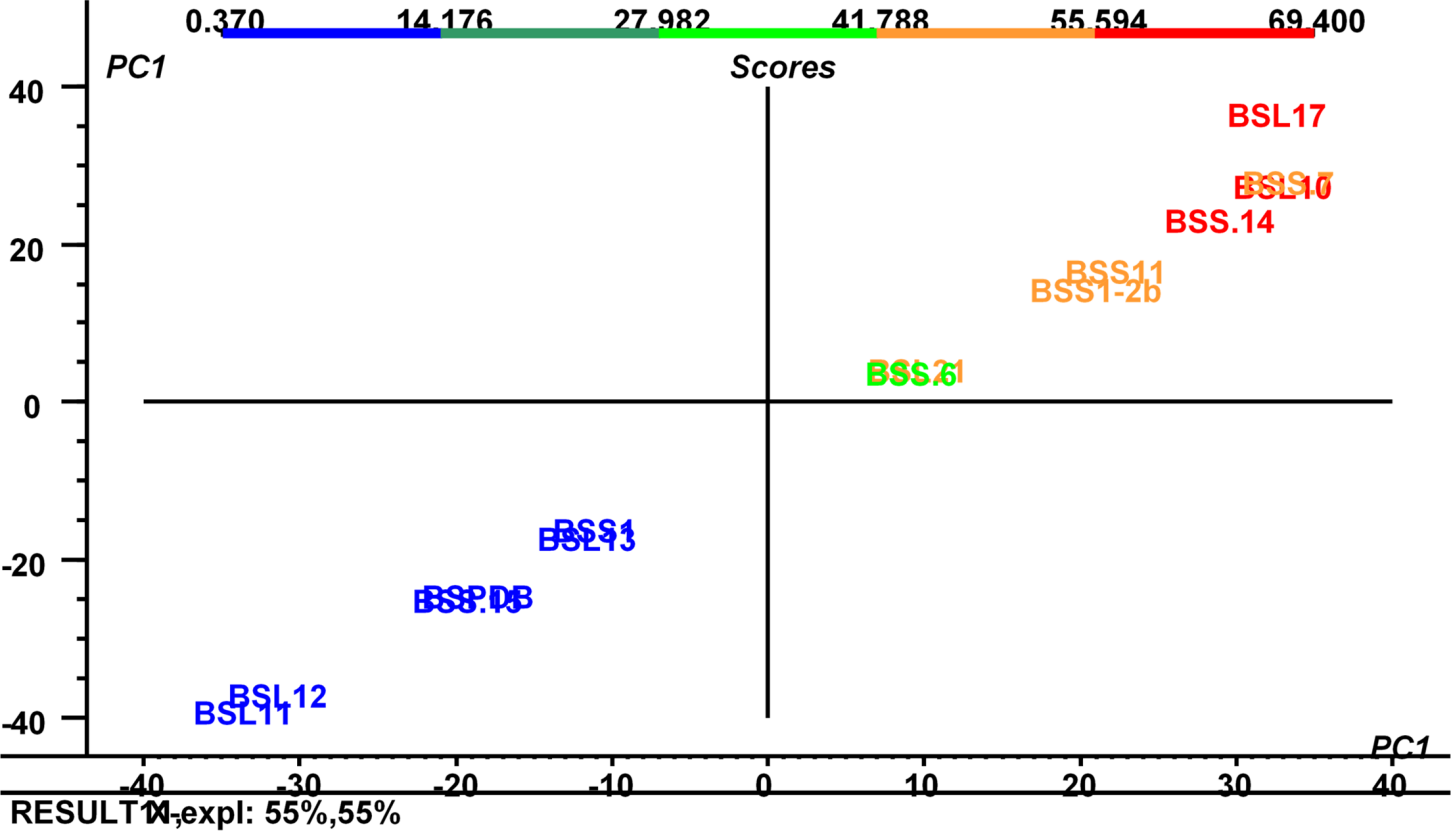
Principal Component Analysis (PCA) analysis. PCA showing the correlation between different endophytic fungi and their enzyme production abilities. In PCA, a full cross-validation was used to validate the enzyme production ability among different species.

The Principal Component Analysis (PCA) plot of different fungal strains also showed similar enzyme production among strains. Three subgroups of fungi can be seen from the PCA plot: BSS11, BSL12, BSS13, BSS5, BSS14, L2, BSL2-1, B3 and BSS7 were observed in group one; BSPDB and BSL11 were observed in group two; and BSL10 and BSS17 were observed in the out-group with a highly significant potential for enzyme production (Fig 4). The two fungi belonged to the same genus of *Preussia* sp., but could be different species, demonstrating quite disparate behavior regarding enzyme production (Table 2).

### Abilities of endophytic fungi to produce indole acetic acid

**Fig 5:**
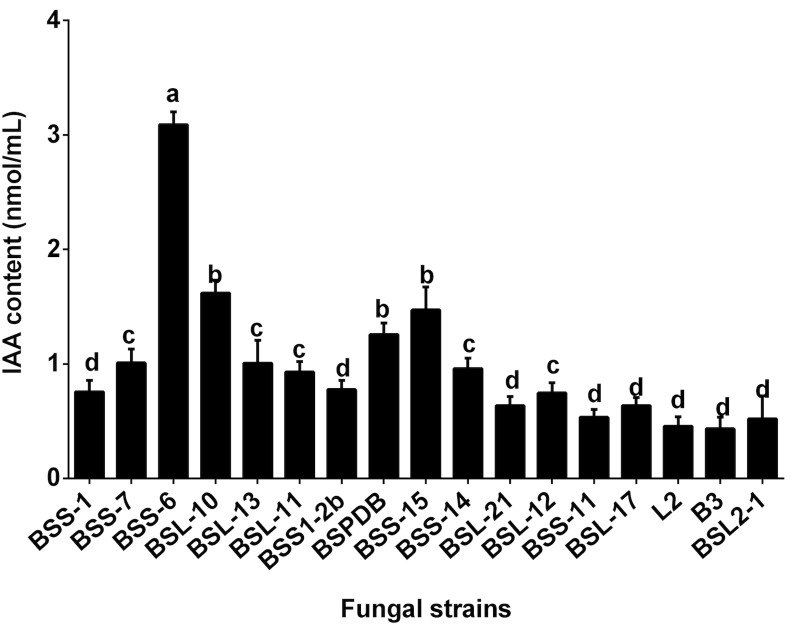
Indole acetic acid (IAA) content in the culture filtrate of various endophytic fungal strains. L-tryptophan-dependent Czapek media was used to grow fungal strains for 7 days. A standard in the same media was also read to yield a standard curve (R^2^ = 0.9985). A total of five replications (100 ml in each Erlenmeyer flask) were used to make sure the validation of IAA results. The bars representing different letters show that the mean values are significantly different from each other, as evaluated by the DMRT test (*P<*0.05).

**Fig 6:**
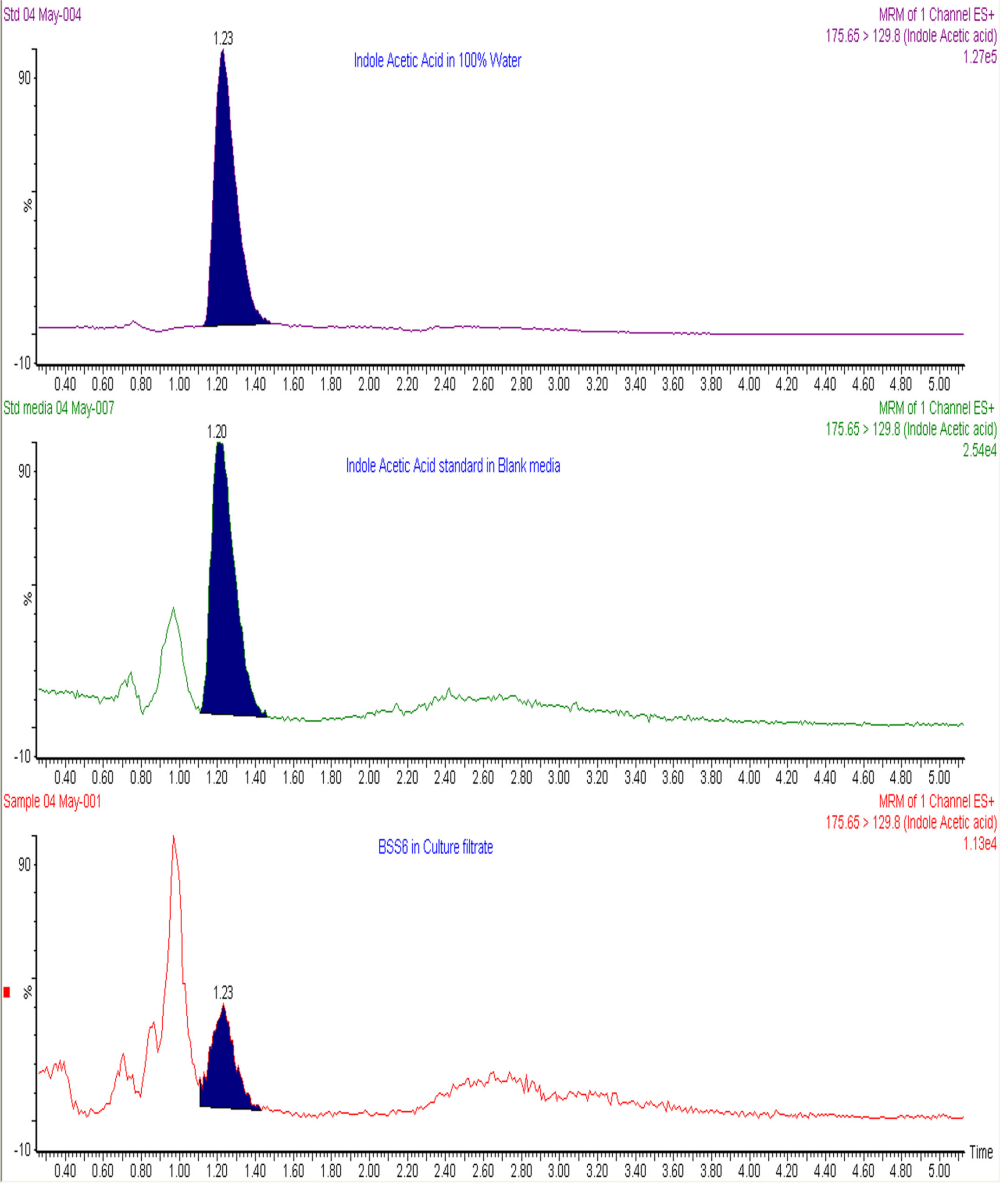
UPLC-MS/MS of IAA. Chromatogram of the culture filtrate of BSS6 (*Aureobasidium* sp.) showing the correlation between the IAA peak and the standard IAA peak in the same fungal growth media and in water.

An advanced chromatographic and mass spectrophotometric method was used to quantify the IAA content in the pure culture filtrates of fungi. Both L-tryptophan-dependent and L-tryptophan-independent pathways were assessed in all fungal strains. In the L-tryptophan-independent pathway, only BSS6 (*Aureobasidium* sp.) showed potential for IAA production, while other fungal strains did not show any potential. In the L-tryptophan-dependent pathways, almost all the fungal strains showed the potential to produce IAA. *Aureobasidium* sp. (BSS6) showed significantly higher IAA synthesis. The results suggested that it synthesizes IAA by both L-tryptophan-dependent and–independent pathways. It was found that BSS6 produces 3.11 ± 0.29 nmol/mL of IAA (Fig 5). This was the highest amount of IAA produced; therefore, an advanced UPLC-MS analysis was performed, in which the presence of IAA was also confirmed in the growth culture media of BSS6. The IAA peaks of the pure culture of BSS6, standard in blank media and standard in 100% water are shown in Fig 6. In other fungal strains, *Preussia* sp. BSL10 and *Aureobasidium pollulan* BSS15 showed significantly higher IAA synthesis. In conclusion, the results suggest that among the 17 isolated endophyte/fungal strains, *Preussia* sp. and *A*. *pollulan* had the ability to produce phytohormones.

### Promotion of the growth of the host tree

**Fig 7:**
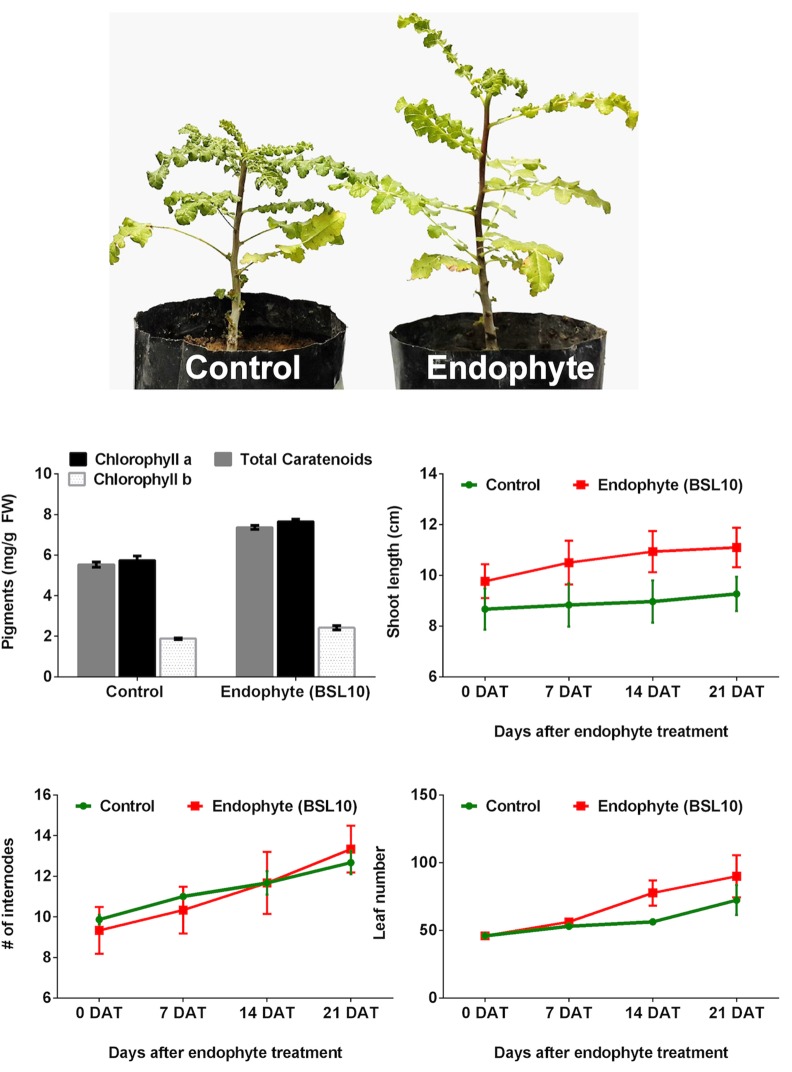
Influence of endophyte inoculation on Host growth. Effect of the application of endophytic fungus (*Preussia sp*. BSL10) on the growth dynamics and photosynthetic pigments of *Boswellia sacra* tree saplings. Each treatment included nine replications. The photosynthetic pigments were measured after 21 DAT. The graphs show the mean value of three replications with standard deviations. 0 DAT shows the readings without any treatments applied.

The four-week-old *B*. *sacra* saplings were inoculated with the endophytic fungus *Preussia* sp. BSL10, which showed an overall highest potential to produce enzymes and IAA production. The results showed that the inoculation of *Preussia* sp. BSL10 to the root zone of the host sapling has significantly (*P*< 0.0001) increased the growth dynamics of *B*. *sacra* plants compared to controls (Fig 7). The results showed that the application of endophytes significantly increased the shoot length (20.12%), internodes (1.1%), and leaves (23.01%) in comparison to controls (Fig 7). This was also confirmed by the observation of significantly increased photosynthetic pigments. The contents of chlorophyll *a*, chlorophyll *b* and total carotenoids were 31.6%, 23.8% and 32.17% higher, respectively, in endophyte-inoculated plants compared to controls (Fig 7). This suggests that host-derived endophytic fungi can influence the growth of the host plant.

## Discussion

*Boswellia sacra*, one of the most culturally and economically important frankincense- or olibanum-producing trees, is not known for its endophytic fungal diversity. The *B*. *sacra* tree has been the focus of scientific attention due to its long history of medicinal uses in Arabic culture. In the current study, the resin producing tree’s bark and leaf parts was selected to explore the associated fungal diversity. Among tree tissues, the fungal-bearing isolates were significantly higher in leaves compared to stems. A similar conclusion was also drawn by Kumar and Hyde [35], Santamaria and Bayman [28,36], Liu et al. [37], Sakayaroj et al. [38], Sun et al. [39], Ghimire et al. [40], Garcia et al. [41], Huang et al. [42], El-Nagerabi et al. [43], and Yan et al. [8], who studied the endophytic fungal diversity of various economically important plants and emphasized on exploring it with ecologically unique plants. A recent report by El-Nagerabi et al. [43] on the endophytic fungal diversity of the frankincense tree of the Dhofar region in Oman also showed that 61% of isolated endophytes exist in the leaf portion. Thus, the endophytic fungal diversity is significantly higher in the leaf relative to the stem. This could be attributed to the compatibility of endophytic organisms in specific tissues and the availability of nutrients to nourish the plant [22]. The endophytic microbial diversity of the frankincense tree was previously reported to be 43 species [38]; combined with the current results, it reached up to 121 endophytic fungal strains in the leaf and stems. However, El-Nagerabi et al. [43] used spore identification method to assess the endophytic fungal population associated with the tree, whereas we used both molecular and morphological methods to understand the fungal communities. This diversity can be even greater if further studies are performed on the rhizosphere of the tree with metagenomic approaches.

In the current study, we identified 5 new species (*Preussia* sp., *Thielavia arenaria*, *Phoma medicaginis*, *Aureobasidium* sp., and *Dothideomycetes* sp.,) of endophytic fungi are reported here for the first time as endophytes from this region. Some of the species, such as *Preussia* sp., are very unique, for instance very few species of this genus have been reported. In the present study, we isolated this species from both the stem and leaf, suggesting its prevalence in the *B*. *sacra* tree and in the Arabic region as shown by Arenal et al. [44]. On the other hand, species of *Dothideomycetes* sp. mostly exist with tree bark as epiphytes or endophytes [45]. We isolated this species from both the leaves and stems. It is known to play an essential role in the degradation of cellulose and leaf litter [45]. We sequenced the fungal ITS region of the rRNA gene following the methods of Arenal et al. [44] and Huang et al. [46], which are more authentic molecular techniques relative to the morpho-taxonomical assays performed in previous studies of the frankincense tree. Phylogenetic analysis further supported our fungal identification methods and diversity [30].

Endophytic diversity has often been presented as a vital necessity for the host plant to combat the unforeseen climatic and environmental changes in the rhizosphere and phyllosphere. Various reports by Waller et al. [47], Rodriguez et al. [48], Khan et al. [13–15], Mercado-Blanco and Lugtenberg [49], and Yan et al. [8] have shown that endophytic symbiosis can help the host in extreme environmental conditions. This potential has also been considered due to the ability of endophytes to produce various types of biologically active metabolites and enzymes. Kusari and Spiteller [12] and Khan et al. [15] suggested that endophytic fungi can produce physiologically active phytohormones, such as gibberellins and auxin, and the ameliorative effects of endophytes are comparable with those of commercially available plant growth regulators (gibberellic acid–GA_3_) during stressful conditions [50]. Based on previous findings, we analyzed the isolated endophytes of the frankincense tree for production of auxin (indole acetic acid–IAA). We hypothesized that endophytes living inside the stems or leaves may also produce such metabolites, which could help the host to survive in harsh environmental conditions.

The analysis of pure fungal CF via UPLC-MS/MS showed that among 17 endophytic strains, only BSS6 (*Aureobasidium* sp.) produced L-tryptophan (Trp)-independent IAA. This was confirmed using both colorimetric and advance chromatographic techniques and authentic standards. A species of *Aureobasidium* sp. has recently been reported by Sun et al. [34] to produce IAA in tryptophan-dependent media using Salkowski reagent. Our results further confirm that *Aureobasidium* sp. can produce IAA by both tryptophan-dependent and -independent pathways. However, UPLC/MS/MS analyses are more sensitive, authentic and reliable. Previous reports also suggest that using advanced techniques for phytohormonal quantification can be more reliable compared to colorimetric methods [46]. *Chaetomium* sp. [14] and *Penicillium citrinum* [51–52] have been reported to produce IAA; however, *Thielavia arenaria*, *Phoma medicaginis*, and *Dothideomycetes* sp., are reported for the first time to have the potential for IAA production. Trp-dependent and Trp-independent IAA biosynthetic pathways reportedly coexist in plants [53] and microbes [54]. However, the majority of previous studies on IAA biosynthesis have evaluated Trp-dependent processes. Few studies have evaluated the Trp-independent pathways of IAA biosynthesis. The intermediates, intermediate stages, and genes involved in Trp-independent pathways have yet to be fully defined in fungi. The fungal IAA biosynthetic pathway has not been widely investigated [50, 55]. In the current study, we elucidated the potential of endophytes/fungi to produce IAA through Trp-dependent and -independent pathways. However, IAA-related mutant/precursor and biosynthetic gene expression analysis could further illustrate the IAA production potential of these strains.

In addition to the ability of endophytes to produce bioactive metabolites, such as IAA, extracellular enzyme production has also been regarded as a resilient feature of endophytes to support the growth of host plant [51]. The *in situ* production of such enzymes in the host by endophytes has not been elucidated fully, but this potential can maintain a continuous supply of adjoining nutrients and resistance mechanisms against pathogenic invasion [17, 56]. The production of extracellular enzymes for the penetration and limited colonization of selected plant cells is a common trait of endophytic fungi [19,56–57]. In the present study, cellulases, phosphatases and glucosidases were quantified using fluorogenic substrates. The current findings showed that species of *Penicillium* and *Preussia* produce significantly higher amounts of cellulases, phosphatases and glucosidases. In the current study, the enzymatic potential of many species, including *Preussia* sp., has been elucidated for the first time. However, other fungi, such as *Penicillium*, have been previously reported to possess a strong potential to produce these enzymes extracellularly [57–58]. *Phoma* sp. is also known for the production of pectinases, cellulases, xylanases, and proteases [59]. However, some of the endophytic fungal strains did not produce large quantities of enzymes in pure CF. This could be due to the physiological variation, adaptation and evolution of fungi, the type of host, the original habitat, and environmental factors [60]. Such hydrolytic enzymes may be suitable for application in the bioconversion of the lignocellulosic biomass into fermentable sugars [56]. An important consideration is the range of substrates that can be utilized by endophytic microorganisms. Studies have shown that endophytes are capable of metabolizing *in vitro* most substrates found in plants, and they can produce enzymes such as proteases, amylases, phenol oxidases, lipases, laccases, polyphenol oxidases, cellulases, mannanases, xylanases, and pectin lyase [8,9,60–62]. The enzymes derived from fungi and bacteria are often more stable than other sources and are used to process raw materials in the food, medicine, beverage, candy, textile and leather industries [63].

The production of such enzymes and hormones by fungal endophytes could play an essential role in the growth and development of the host plant. In the current study, the application of *Preussia* sp. BSL10 to the host *B*. *sacra* saplings appreciably increased the shoot length, leaf number, internodes and quantities of photosynthetic pigments (chlorophyll *a*, *b* and total carotenoids). This is in agreement with previous reports, which showed that endophytic inoculation [*Paecilomyces formosus* LHL10, *Penicillium janthinellum* LK5, and *P*. *citrinum*] to the host plants resulted in the improvement of growth and stress tolerance [14, 16, 21, 51]. These studies along with many others have suggested that endophytic inoculation in the root zone can improve physiology and development of phyllospheric parts (leaf, stem and seeds) of the plants [64]. Findings of Ritpitakphong et al. [65] showed that inoculation of leaf microbiome could improve tolerance of plant against pathogenic attacks. A similar conclusion was drawn when fungal endophyte *Penicillium resedanum* LK6 was applied to *Capsicum annuum* L. and it improved not only the growth of pepper fruits but also increased the functional biochemical (capsaicin) contents during drought stresses [66]. This is contributed to the ability of endophytes in the production of gibberellins and indole acetic acid, which are the known plant growth regulating hormones and also commercially utilized for crop improvement in marginal lands [22, 67]. In addition, the endophytic association might also intervene the mineral relocation and enzyme production, that in-turns benefits the biomass construction in the aerial part of the plant [68].

In conclusion, the enzymes produced by fungal endophytes inside host tissues could help in supplementing the direct uptake of nutrients by microorganisms in the roots. Endophytes have the potential to secrete extracellular enzymes (cellulases, phosphatases and glucosidases) and plant-based hormones (such as auxin), which can help the host in harsh environmental conditions and also strengthen the symbiotic bond between the endophyte and the host. The present elucidation of endophytic diversity from *Boswellia sacra* grown in extreme drought conditions indicates that these contributing factors stimulate host growth. A similar prospect was also found for the wild trees of the Amazon basin, which are abundant with endophytic fungal diversity. Such endophytic diversity has been suggested to enhance mutualism and protect the host against insect attacks [22, 57].

## Supporting Information

S1 Table Extracellular enzymes and substrates. List of fluorescence enzymes, their substrates and products.
(DOCX)

S2 Table Conditions for Indole Acetic Acid quantification. Details of UPLC-MS/MS conditions used to analyze the content of Indole Acetic Acid in the pure culture of endophytic fungus.
(DOCX)

S1 Fig Standard curve reading of enzymes. Standard curve readings of 4-methylumbelliferone (MUB,) vs. the standard substrate using florescence spectrophotometry.
(DOCX)

S2 Fig Fungal endophytes isolated. Endophytes from different phyllospheric parts of the frankincense tree.
(DOCX)
