# Supplementary material for: Endophytic Fungi from Frankincense Tree Improves Host Growth and Produces Extracellular Enzymes and Indole Acetic Acid
Source: PLoS One. 2016 Jun 30;11(6):e0158207. doi: 10.1371/journal.pone.0158207 (PMC4928835; doi:10.1371/journal.pone.0158207)
Supplement: S1 Table — List of fluorescence enzymes, their substrates and products. (DOCX) [file pone.0158207.s001.docx]

**S1 Table.** **Extracellular enzymes and substrates.** List of fluorescence enzymes, their substrates and products.

| **Enzymes** | **Function** | **Florigenic substrate** | **Concentration** |
| --- | --- | --- | --- |
| β-1,4-glucosidase | Cellulose to glucose | 4-MUB- β -D-glucopyranoside  3.2.1.21 | 10 - 100 μM |
| 1,4- β -cellobiosidase | Cellulose to disaccharide | 4-MUB- β -D-cellobioside  3.2.1.91 | 10 - 100 μM |
| Phosphatase | Phosphomonoesters  to phosphate | 4-MUB-phosphate  3.1.3.1 | 10 - 100 μM |

MUB = methylumbelliferone
