# Supplementary material for: Endophytic Fungi from Frankincense Tree Improves Host Growth and Produces Extracellular Enzymes and Indole Acetic Acid
Source: PLoS One. 2016 Jun 30;11(6):e0158207. doi: 10.1371/journal.pone.0158207 (PMC4928835; doi:10.1371/journal.pone.0158207)
Supplement: S2 Table — Details of UPLC-MS/MS conditions used to analyze the content of Indole Acetic Acid in the pure culture of endophytic fungus. (DOCX) [file pone.0158207.s002.docx]

**S2 Table. Conditions for Indole Acetic Acid quantification.** Details of UPLC-MS/MS conditions used to analyze the content of Indole Acetic Acid in the pure culture of endophytic fungus

| **UPLC conditions for IAA analysis** | |
| --- | --- |
| Equipment | Ultra Performance Liquid Chromatography (UPLC) system (Acquity UPLC, Waters Inc. USA) |
| Column | Waters Acquity BEH HILIC (2.1 X 50 mm 1.7 µm) analytical column |
| Column oven Temperature | 40°C |
| Injection volume/mode | 10 μL/ partial loop with needle overfill (PLUNO, Waters Inc. USA) |
| Flow rate of Solvent A, B | 0.2 ml·min^-1^ (Channel A=0.5% formic acid in water; Channel B= 0.5% formic acid in acetonitrile) |
| Gradient (A%/B%) | (95%/5%) 3 min → (10%/90%) 1 min → (introduced starting composition) 4.1 min → (pre-equilibration time) 1.9 min→ (total analysis time) 6 min |

| **MS/MS conditions for IAA analysis** | |
| --- | --- |
| Equipment | Waters Quattro Premier XE Tandem Quadrupole system (Waters Inc. USA) |
| Tuning parameters optimization | 4 ng/µL (ppm) indole acetic acid |
| Source operation parameters during electrospray ionization (ESI+) |  |
| Capillary voltage | 3.0 kV |
| Cone voltage | 12.0 V |
| Collision Energy | 13 eV |
| Source temperature | 150 °C |
| Desolvation temperature | 400°C |
| Desolvation gas | 1000 L h^-1^ |
| Cone gas | 20 L h^-1^ |
| Precursor/ product ion optimization | 175.65/ 129.8 |
