# Supplementary material for: Endophytic Fungi from Frankincense Tree Improves Host Growth and Produces Extracellular Enzymes and Indole Acetic Acid
Source: PLoS One. 2016 Jun 30;11(6):e0158207. doi: 10.1371/journal.pone.0158207 (PMC4928835; doi:10.1371/journal.pone.0158207)
Supplement: S1 Fig — Standard curve readings of 4-methylumbelliferone (MUB,) vs. the standard substrate using florescence spectrophotometry. (DOCX) [file pone.0158207.s003.docx]

**
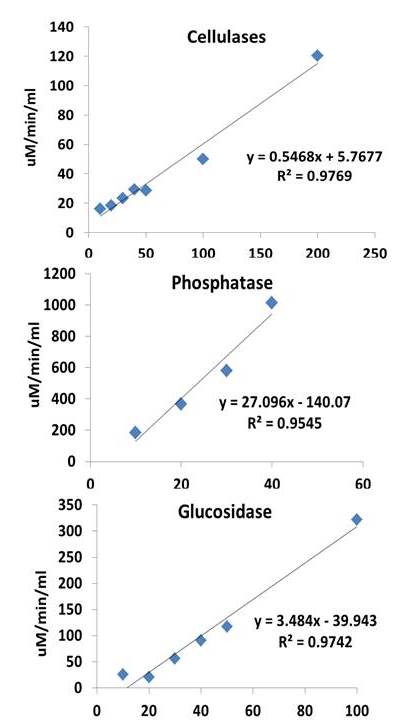
**

**S1 Fig. Standard curve reading of enzymes.** Standard curve readings of 4-methylumbelliferone (MUB,) vs. the standard substrate using florescence spectrophotometry.
