# Supplementary material for: Endophytic Fungi from Frankincense Tree Improves Host Growth and Produces Extracellular Enzymes and Indole Acetic Acid
Source: PLoS One. 2016 Jun 30;11(6):e0158207. doi: 10.1371/journal.pone.0158207 (PMC4928835; doi:10.1371/journal.pone.0158207)
Supplement: S2 Fig — Endophytes from different phyllospheric parts of the frankincense tree. (DOCX) [file pone.0158207.s004.docx]

**
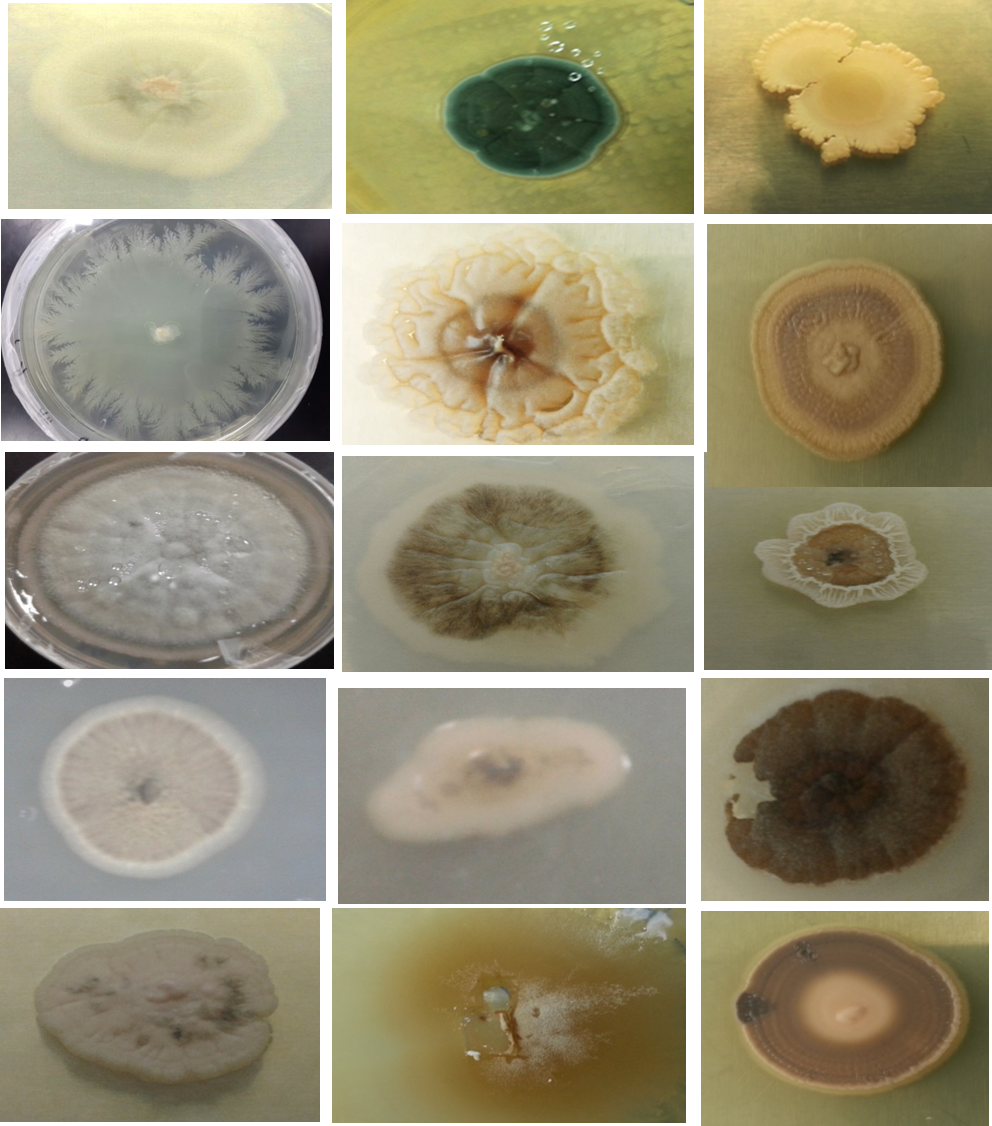
**

**S2 Fig. Fungal endophytes isolated**. Endophytes from different phyllospheric parts of the frankincense tree.
